# Die Zukunft der interventionellen Radiologie: mehr als nur Bildgebung

**DOI:** 10.1007/s00117-025-01534-x

**Published:** 2025-11-04

**Authors:** Mohammed Bahaaeldin, Daniel Kütting

**Affiliations:** Radiologische Klinik, Universitätsklinikum Bonn, Venusberg-Campus 1, 53127 Bonn, Deutschland

## Lernziele.

- Die Rolle evidenzbasierter Medizin in der Weiterentwicklung der IR verstehen
- Den Einfluss von KI, Robotik und VR auf IR-Workflows und Ausbildung einschätzen
- Die Bedeutung der Ambulantisierung für die IR-Versorgung erfassen
- Argumente für und gegen die organisatorische Eigenständigkeit der IR abwägen

Die interventionelle Radiologie (IR) hat sich von einem bildgebungsnahen Dienstleister zu einer eigenständigen, therapeutischen Disziplin entwickelt. Sie übernimmt zunehmend die Rolle eines aktiven Behandlers, der minimal-invasive, evidenzbasierte Therapien anbietet. Ihr Spektrum reicht von Embolisationen und Ablationen bis zu komplexen Rekonstruktionen über alle Organsysteme hinweg – stets gestützt durch modernste Bildgebung.

Parallel wächst die wissenschaftliche Evidenzbasis: multizentrische, randomisierte Studien adressieren objektivierbare, Outcome-relevante Endpunkte. Diese Daten ermöglichen die Integration der IR in Leitlinien und etablieren sie als festen Bestandteil standardisierter Therapiekonzepte. Damit positioniert sich die IR als integrale, evidenzbasierte Säule der modernen Medizin.

## Evidenzbasierte Behandlung

Die Evidenzbasis für interventionell-radiologische Verfahren entwickelt sich zunehmend von Beobachtungs- zu randomisierten, multizentrischen Studien. Diese Daten sind entscheidend, um Verfahren aus der Einzelfallentscheidung herauszuführen und ihre Anwendung evidenzbasiert in Leitlinien und Kostenträgerentscheidungen zu verankern. Erforderlich sind Studien, die objektivierbare und patientenrelevante Endpunkte adressieren – insbesondere Gesamtüberleben („overall survival“, OS), progressionsfreies Überleben (PFS), Funktionsverbesserung und Lebensqualität – sowie transparente Vergleichsarme und ein ausreichendes Follow-up bieten.

In der interventionellen Onkologie verschiebt sich der Fokus von reiner Lokaltherapie hin zu multimodalen Konzepten. Bei hepatozellulärem Karzinom werden Chemoembolisation (TACE) und Radioembolisation zunehmend in immunonkologische Therapieschemata integriert. Bildbasierte Biomarker und quantitative Tumorlast-Scores ermöglichen eine präzisere Patientenselektion. Phase-III-Studien wie EMERALD‑1 belegen, dass TACE eine tragende Säule multimodaler Therapien ist – nicht nur als palliative Lokaltherapie, sondern als integraler Bestandteil krankheitsmodifizierender Kombinationstherapien [1]. Die COLLISION-Studie belegte die Gleichwertigkeit der Ablation zur chirurgischen Resektion bei kolorektalen Lebermetastasen, wodurch Ablation zunehmend als organschonende Erstlinienoption diskutiert wird [2].

Auch in nichtonkologischen Indikationen wird die Evidenzbasis zunehmend breiter: Für die Uterusarterienembolisation (UAE) liegen mittlerweile Langzeitdaten vor, die den Uteruserhalt und eine nachhaltige Symptomkontrolle belegen und damit die UAE als etablierte Alternative zur Hysterektomie [3] in Leitlinien verankert haben. Neue Verfahren wie die genikuläre Arterienembolisation bei Gonarthrose, die aktuell in randomisierten Studien evaluiert wird, verdeutlichen zusätzlich das Potenzial, den Gelenkprothesenersatz hinauszuzögern und interventionell-radiologische Verfahren als eigenständige Therapieoptionen weiter zu etablieren.

Auch im Bereich venöser Interventionen wird Evidenz geschaffen: Die randomisierte, multizentrische PEERLESS-II-Studie prüft, ob mechanische Thrombektomie zusätzlich zur Antikoagulation bei intermediär-riskanter Lungenembolie einen Vorteil gegenüber Antikoagulation allein bietet [4]. Ihre Ergebnisse werden maßgeblich die zukünftige Leitlinienempfehlung bestimmen.

## MR-gesteuerte Interventionen

Neben klassischen CT- und Ultraschall-gestützten Verfahren gewinnen MR-gesteuerte Interventionen zunehmend an Bedeutung. MR-Mammabiopsien sind bereits fester Bestandteil des klinischen Alltags, doch technologische Weiterentwicklungen eröffnen neue Einsatzfelder. Besonders relevant sind moderne MRT-Systeme mit niedrigem Magnetfeld (< 1 T) und erweiterter Gantry-Öffnung, die den Patientenzugang erleichtern und trotz reduzierter Feldstärke eine ausreichende Bildqualität bieten. Dadurch werden Eingriffe möglich, die bislang als technisch limitierend galten.

Ein zentrales Anwendungsfeld sind MR-gesteuerte Lebertherapien und Biopsien. Die hohe Weichteilkontrastierung und multiplanare Bildführung ermöglichen eine präzise Zielpunktauswahl und damit eine gesteigerte Treffsicherheit bei kleinen oder schwer zugänglichen Läsionen [5]. In der Therapie erlauben MR-gesteuerte Verfahren – etwa Ablationen – eine exakte Platzierung der Applikatoren sowie eine unmittelbare Kontrolle der thermischen Ausdehnung in Echtzeit, was die Sicherheit und Vollständigkeit des Eingriffs verbessert. Die strahlungsfreie Durchführung ist ein zusätzlicher Vorteil, besonders bei jüngeren Patienten oder bei wiederholten Prozeduren.

Die Perspektiven gehen über einzelne Eingriffe hinaus: Durch die Integration funktioneller Bildgebung (z. B. Perfusion, Diffusion) können Therapien zunehmend biologisch gesteuert werden, etwa durch gezielte Behandlung vitaler Tumoranteile. Erste Phase-I/II-Studien berichten über eine hohe technische Erfolgsrate und vielversprechende lokale Kontrollraten für MR-gesteuerte Ablationen [6]. Diese Daten stützen den Weg in Richtung personalisierte, präzisionsmedizinische Therapieplanung und werden voraussichtlich in zukünftige Leitlinien einfließen.

## Künstliche Intelligenz, Robotik und Virtual Reality

Künstliche Intelligenz (KI) findet zunehmend Anwendung – auch in der IR – und unterstützt den gesamten Workflow von der Triage über die Interventionsplanung bis zur Qualitätssicherung. Derzeit dominieren Assistenzfunktionen wie automatische Segmentierung, Läsionscharakterisierung und dosisoptimierte Bildrekonstruktion [7]. Neue Entwicklungen gehen weit darüber hinaus:

*Workflow-Optimierung*: KI-Systeme können Interventionsanfragen automatisch priorisieren und an die zuständigen Teams routen, was die Ressourcensteuerung verbessert und Wartezeiten reduziert.

*Bildregistrierung und Navigation*: Deep-Learning-Modelle ermöglichen die Fusion von CT-, MR- und Ultraschalldaten in Echtzeit und verbessern so die Zielgenauigkeit bei komplexen Punktionen oder Ablationen [8].

*Instrumentenerkennung:* KI-Algorithmen können Katheter, Drähte oder Ablationssonden in Durchleuchtungsbildern automatisch identifizieren und ihre Position dreidimensional rekonstruieren, auch bei niedriger Dosis, was die Sicherheit erhöht.

Intraprozedural können KI-gestützte Overlays und registrierte Volumina die Präzision steigern. Robotische Assistenzsysteme nutzen zunehmend lernbasierte Steuerungen, um Organbewegungen zu kompensieren und Nadeln automatisch zu positionieren. Studien zeigen Verbesserungen bei Zielgenauigkeit, Lernkurve und Strahlenexposition, ohne Sicherheitsnachteile [9].

Extended-Reality-Technologien (XR) – inklusive Virtual und Augmented Reality (AR) – werden für präprozedurale Planung, Simulation komplexer Zugangswege und intraoperative Navigation genutzt. Patientenspezifische 3D-Modelle unterstützen die Auswahl optimaler Katheter- oder Ablationspfade, und AR-Overlays visualisieren anatomische Landmarken in Echtzeit [10]. Für die Ausbildung ermöglichen XR-Simulatoren standardisierte Trainingsszenarien, während erste Studien auch eine Reduktion von Angst und Schmerzempfinden bei Patienten zeigen.

Für eine breite Implementierung sind prospektive Studien mit klar definierten Metriken (Trefferquote, Prozedurzeit, Komplikationsrate, Kontrastmittelverbrauch) notwendig. Ausbildungsprogramme sollten Kompetenzen in Datenwissenschaft und Medizintechnik integrieren, um die sinnvolle Nutzung von KI-gestützten Systemen zu gewährleisten.

### Ambulantisierung

Die ambulante und tagesstationäre Versorgung gewinnt in der IR zunehmend an Bedeutung. Treiber sind Patientenzentrierung, Effizienzsteigerung, Kapazitätsengpässe im stationären Bereich und der „Less-is-more-Vorteil“ minimal-invasiver Verfahren. Richtig implementiert tragen ambulante Pfade zu den Zielen des Triple-aim-Ansatzes bei: kürzere Aufenthalte, geringere Morbidität und Kosten sowie eine verbesserte Patientenerfahrung.

#### Für eine sichere Ambulantisierung sind wesentliche Bausteine erforderlich:

- Stringente Patientenselektion (z. B. ASA-Klasse, Komorbiditäten, soziales Umfeld)
- Standardisierte Sedierungs- und Analgesiepfade
- Klare Entlassungskriterien und strukturierte Nachsorge (inkl. Telemedizin)
- Definierte 24/7-Rückfallebene und eindeutige Eskalationswege

Geeignete Prozeduren sind u. a. Portimplantationen, ausgewählte Embolisationen, perkutane Biopsien und Ablationen, Drainagen und viele gefäßinterventionelle Eingriffe ohne Hochrisikoprofil. Parallel verlagert sich die Diagnostik näher an den Patienten („Home-Hospital-Konzepte“), und Workflows werden zunehmend krankheits- statt standortgetrieben organisiert.

Ökonomisch werden Hybrid-DRG-Modelle wichtiger, da sie ambulante und stationäre Leistungen einheitlich abbilden. Die Erweiterungen der Hybrid-DRG-Kataloge 2024/2025 zeigen, dass immer mehr IR-Prozeduren sektorenübergreifend abrechenbar werden. Für die IR sind belastbare Vergütungslogiken, Evaluations- und -Managementstrukturen sowie transparente Dokumentations- und Qualitätsnachweise Voraussetzung, damit Aufklärung, Indikationsstellung und Follow-up adäquat honoriert werden. „Outpatient-based laboratories“ (OBLs) und hybride Modelle können Wachstum ermöglichen [11], wenn Qualität, Komplikationsmanagement und Interoperabilität mit Krankenhausstrukturen sichergestellt sind.

## IR als eigenes Fach?

Die Frage nach der organisatorischen Eigenständigkeit begleitet die IR seit Jahren. In den USA ist die IR bereits als eigenständige Facharztausbildung anerkannt. Pro-Argumente sind die kliniknahe Arbeitsweise mit eigenen Sprechstunden, Konsilen, stationärer Mitbetreuung und strukturiertem Langzeit-Follow-up. Eine eigenständige IR erfordert agile Ressourcensteuerung (Personal, Budget, Technik) und flexible Strukturen; exklusive Verträge oder Abhängigkeiten von bildgebungsgetriebenen Erlösmodellen können die Entwicklung hemmen. Zudem unterscheiden sich Kultur und Arbeitsweise deutlich von rein diagnostischen Bereichen [12].

Gleichzeitig bleibt die enge Verbindung zur diagnostischen Radiologie fachlich essenziell: Bildkompetenz ist ein Kernwert der IR, und gemeinsame Ausbildungspfade haben ihre Professionalisierung maßgeblich gefördert.

Realistisch erscheint daher ein hybrides Modell: organisatorisch eigenständige IR-Einheiten mit klarer klinischer Verantwortung, eng verzahnt mit der diagnostischen Radiologie und interdisziplinären Boards. Ausbildungscurricula sollten verpflichtend Klinikzeit, Notfall- und Stationsarbeit, Abrechnungssysteme, Ressourcen- und Materialkostenmanagement, Qualitätsmanagement und Forschungskompetenz (Studien, Register, Datenanalyse) abbilden. Entscheidend ist weniger die formale Trennung als eine sichtbare klinische Präsenz und Übernahme von Ergebnisverantwortung.

## Fazit für die Praxis

- Die interventionelle Radiologie entwickelt sich von einer bildgebungsnahen Prozedur zu einer klinisch verantwortlichen, evidenzbasierten Therapieplattform.
- Ihre Zukunft wird geprägt durch randomisierte, multizentrische Studien, den Einsatz digitaler Assistenzsysteme (Künstliche Intelligenz, Robotik, Extended Reality) und die Evaluation anhand klinisch relevanter Outcome-Metriken.
- Ergänzend gewinnen strukturierte, sichere ambulante Versorgungspfade mit klarer Patientenselektion und angemessener Vergütung an Bedeutung.
- Ob als eigenständiges Fach oder eng in die Radiologie integriert – entscheidend bleibt der nachweisbare Nutzen für Patientinnen und Patienten.
